# A practical adaptive moving-mesh algorithm for solving unconfined seepage problem with Galerkin finite element method

**DOI:** 10.1038/s41598-019-43391-4

**Published:** 2019-05-06

**Authors:** Qianwei Dai, Yi Lei, Bin Zhang, Deshan Feng, Xun Wang, Xiaobo Yin

**Affiliations:** 1 School of Geosciences and Info-Physics, Central South University, Changsha, 410083 P.R. China; 2 Key Laboratory of Metallogenic Prediction of Nonferrous Metals and Geological Environment Monitoring, Ministry of Education, Changsha, P.R. China

**Keywords:** Hydrology, Fluid dynamics

## Abstract

One of the great challenges of unconfined seepage through a dam lies in the accurate determination of free surface that depends on the complexity of the seepage model, especially if the model is characterized with complex geometry and sharp variations in permeability distribution. This study presents a practical methodology that combines the adaptive moving-mesh algorithm and the Galerkin finite element method (FEM) to solve an unconfined seepage problem with high efficiency and precision. The methodology employs a set of improvement terms, such as remainder factor, step-size parameter and termination condition, all of which guarantee that the simulation and the refinement fitting can be implemented efficiently until the free surface converges within a given allowable error. In particular, a specialized discussion is presented for the significant relation between the location of the exit point and the corresponding grid fineness. To validate the practicability of the proposed method, a series of examples are performed. Comparing the result with those of other numerical approaches, we conclude that even though the unconfined seepage model may be complicated with arbitrary complex geometry and sharp variations in permeability distribution, the proposed algorithm provides a great improvement in efficiency and accuracy in free-surface searching.

## Introduction

Analysis of free-surface seepage problems has been attracting more attention in the past few decades due to its wide variety of scientific and engineering applications, such as geotechnical slopes, earth dams, underground caves, and groundwater movement, and is also beneficial for analysing the interaction and coupling between seepage field and stress field^1,2^. To date, one of the most difficult challenges in solving the unconfined seepage problem is the determination of the free surface of unconfined seepage flow, which still challenges the reinforcement and treatment of dam seepage. Many previous experimental studies have been conducted to investigate the free surface analysis of unconfined seepage problems with different classical numerical approaches^3,4^.

Since the residual flow method and the variational inequality methods were introduced^5–7^, significant improvement has been made with respect to the fixed grid method^8–13^. Desai and Li^5^ analysed the unconfined seepage problem with a fixed mesh technique using a relaxation-type iterative algorithm, called the residual flow method. The main advantage of this technique lies in its capability of treating porous (soil) media with arbitrary inhomogeneity. Bathe and Khoshgoftaar^10^ proposed the unit osmotic matrix adjustment method, which employs a non-linear pressure-dependent permeability description of the material, and Newton-Raphson iterations to avoiding iterations with the finite element mesh. The results demonstrated that this technique is highly effective in analysing free surface flow problems by means of transient seepage analysis. García-Ruíz and Steven^11^ conducted a study to investigate the computational consumption of using the fixed grid finite element method (FEM) and the classical FEM, respectively, and the comparisons results revealed that the fixed method provides a great improvement in saving calculation resources. Zheng *et al*.^12^ gave a new variational inequality formulation to the saturated zones and no-flow zones. The new formulation imposed a boundary condition of Signorini’s type on the potential seepage boundary and eliminated the singularity of the seepage point, upon which the seepage point turned out to be a point that makes both inequalities in Signorini’s complementary condition become equalities. Daneshmand and Kazemzadeh-Parsi^13^ modified the fixed-grid FEM with a new approach for computing the stiffness matrix of boundary intersecting elements. The findings confirmed that, besides the high accordance with the analytic solutions and other numerical solutions, the proposed method possesses the unique advantages of high accuracy and effective convergence.

These approaches more or less modified the potential of each node by means of calculating the seepage discharge that flowed through the free surface, and executing a sustained calculation process until the discharge was less than a certain given value. That is to say, the common characteristic of these approaches, whether fixed-grid or modified fixed-grid method, is the uniform grid chosen that formulates the boundary intersecting elements. As the exact position of the free surface is interpolated between the different computational nodes, in most case, the calculated results of the free surface cannot be presented as a smooth curve.

Another alternative approach is the conventional adaptive mesh method (AMM), which calculates and meshes the domain underneath the free surface. The changes in a variable domain can be conveniently confirmed with an adaptive mesh technique by relocating the boundary elements through successive iterations. This distinctive characteristic makes it one of the most suitable methods for free surface analysis. Starting with the research of Taylor and Brown^14^, Finn^15^, Neuman and Witherspoon^16^, great improvement has been made concerning the seepage modelling technique with the AMM^17–21^. Based on the previous studies and the mass conservation equations, Bardet and Tobita^17^ derived finite difference (FD) equations by a flux conservation. The greatest contribution is that it does not require the formation and reformation of a global matrix system to solve a nonlinear system of equations. Inevitably, it would encounter challenges in enforcing the boundary conditions that result from the complicated shape of the domain. Darbandi *et al*.^19^ proposed the moving-mesh finite volume method (FVM) by assuring a mass conservation over modelling cells, it revealed that, regardless of the type of mesh strategies utilized (i.e. fixed-mesh or moving-mesh), the calculation accuracy with FVM is substantially improved.

In the present work, we focus solely on economic and accurate solutions to handle complicated model cases, and present a practical and innovative methodology that combines the Galerkin FEM with the AMM, where the main parameters of seepage area can all be calculated at each node (e.g. water head, hydraulic gradient, and seepage velocity). In particular, for further improving fitting accuracy and convergence rate, an irregular-mesh scheme of quadrangle grids, as well as a new step-size parameter *λ* and a novel termination factor *ξ*, are introduced in the fitting process of upper surface boundary selection, and as a result, the calculation area is reduced dramatically. It is worth noting that the impact of the unsaturated region on the search results can also be eliminated.

## Results and Discussion

**Figure 1:**
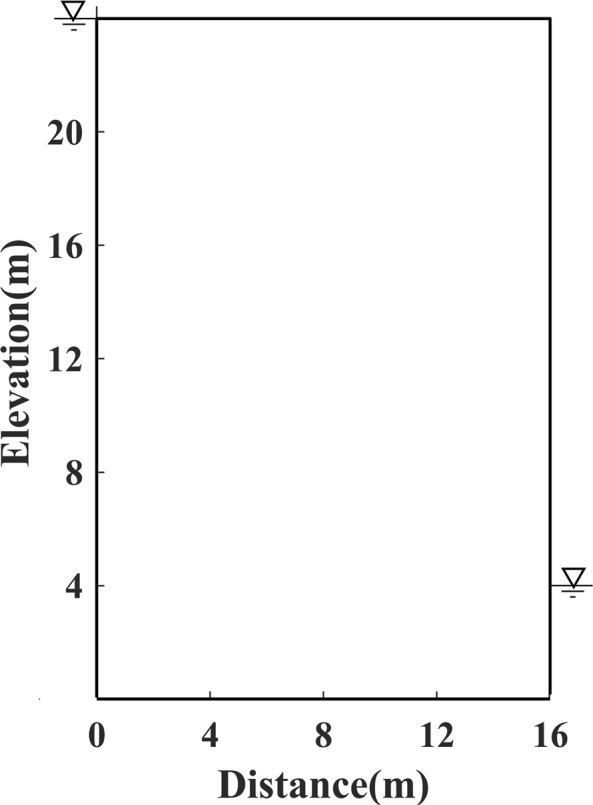
Schematic diagram of Model 1.

**Figure 2:**
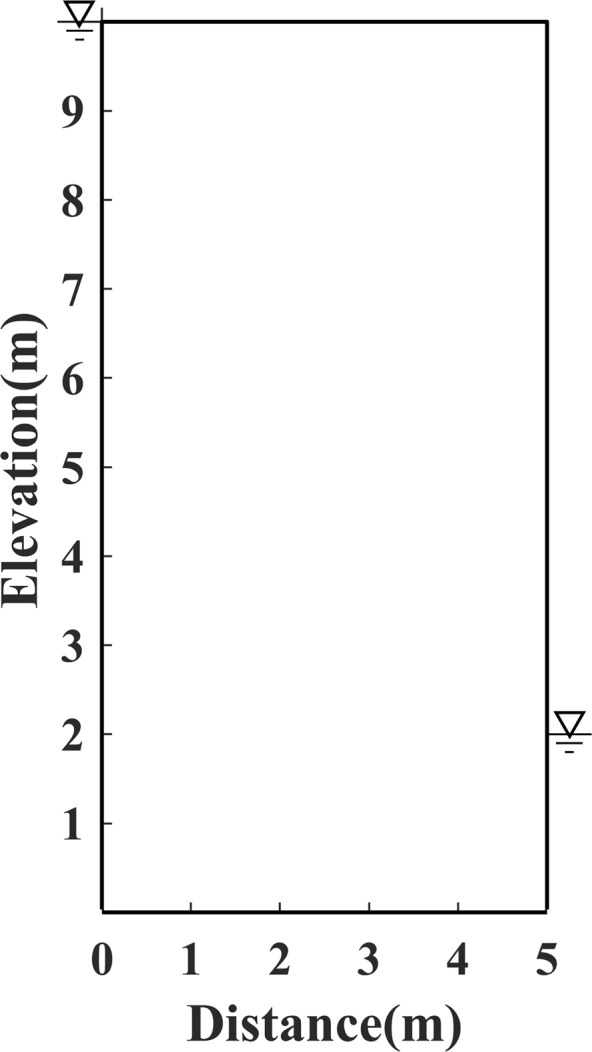
Schematic diagram of Model 2.

**Figure 3:**
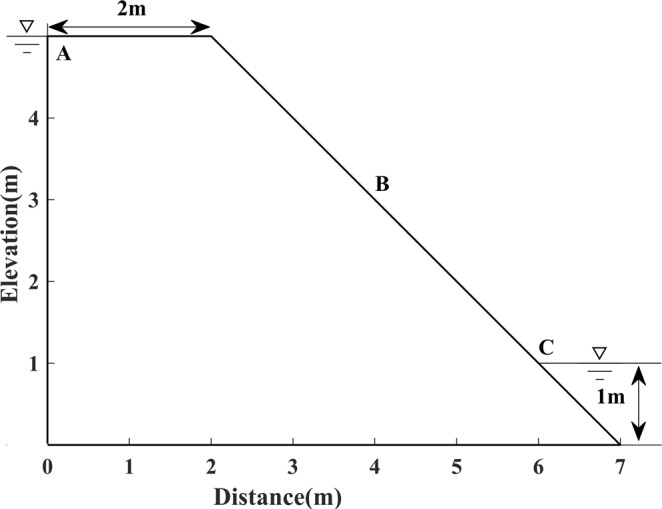
Schematic diagram of Model 3.

**Figure 4:**
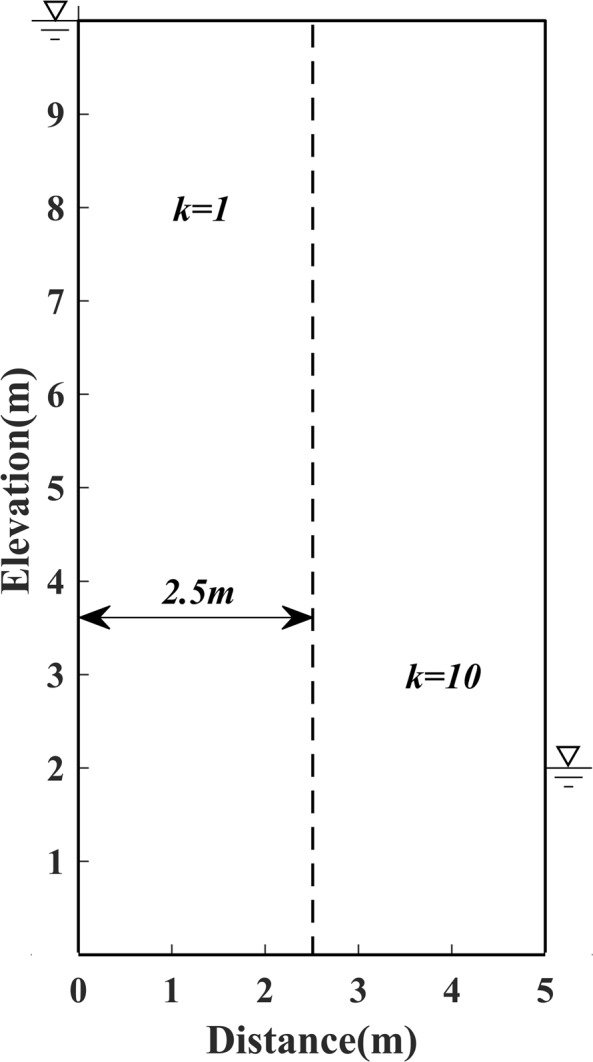
Schematic diagram of Model 4.

The experiments were conducted with four models, e.g. Model 1, the first standard model, is presented as an earth dam 26 metres high and 16 metres wide, the water head is 24 metres high in the upstream reservoir, and 4 metres high in the downstream pool, respectively, as shown in Fig. 1; Model 2, which is established as an earth dam 10 m high and 5 m wide, the head of water is 10 m high in the upstream reservoir, and 2 m high in the downstream pool, as shown in Fig. 2; Model 3, the irregular model, is constructed as an earth dam with a slanted downstream surface, the water head is set to 5 m high in the upstream reservoir, and 1 m high in the downstream pool, as shown in Fig. 3; Model 4, an inhomogeneous media model, is constructed with different permeability distributions in each side of two rectangular vertical blocks (i.e. upstream and downstream vertical block), as shown in Fig. 4.

### Model 1, the first standard model

In this study, a mesh distribution with 64 × 128 grids are chosen as a default distribution to solve the free surface problem by comparing the result with that of the measured data and the other numerical solutions.

In order to highlight the characteristics of different approaches, the most sensitive part of the solution (i.e. the free surface and exit point position) is selected to present the comparison results.

**Figure 5:**
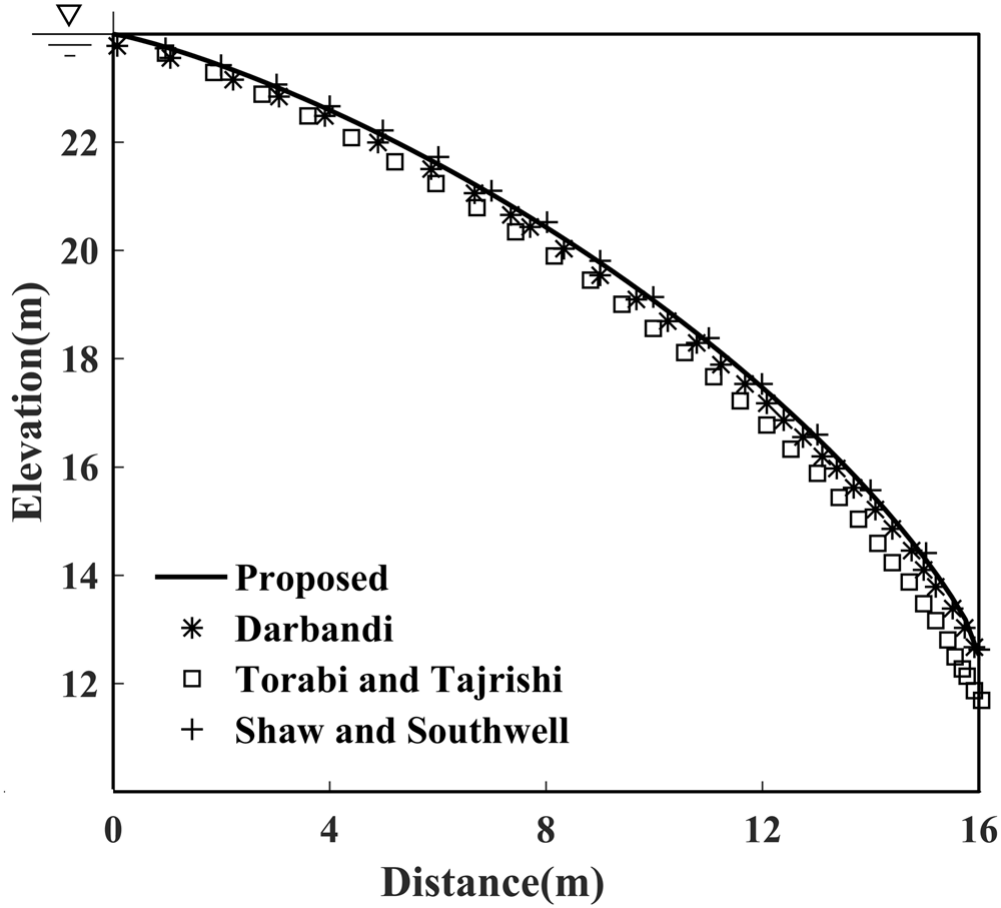
Solutions of free surface for Model 1 calculated by the proposed method.

As shown in Fig. 5, the position of free surface calculated by the proposed method has good coincidence with the measurement data by Shaw and Southwell^22^, the FVM by Darbandi *et al*.^19^, and the FD method by Torabi and Tajrishi^23^. In addition, it revealed that, except for the FD method by Torabi and Tajrishi^23^, the position of free surface along with the exit point agree well both with the measured data and the other numerical solutions. Hence, it can be concluded that the proposed method suitably predicts the exact position of free surface, as well as the exit point.

**Figure 6:**
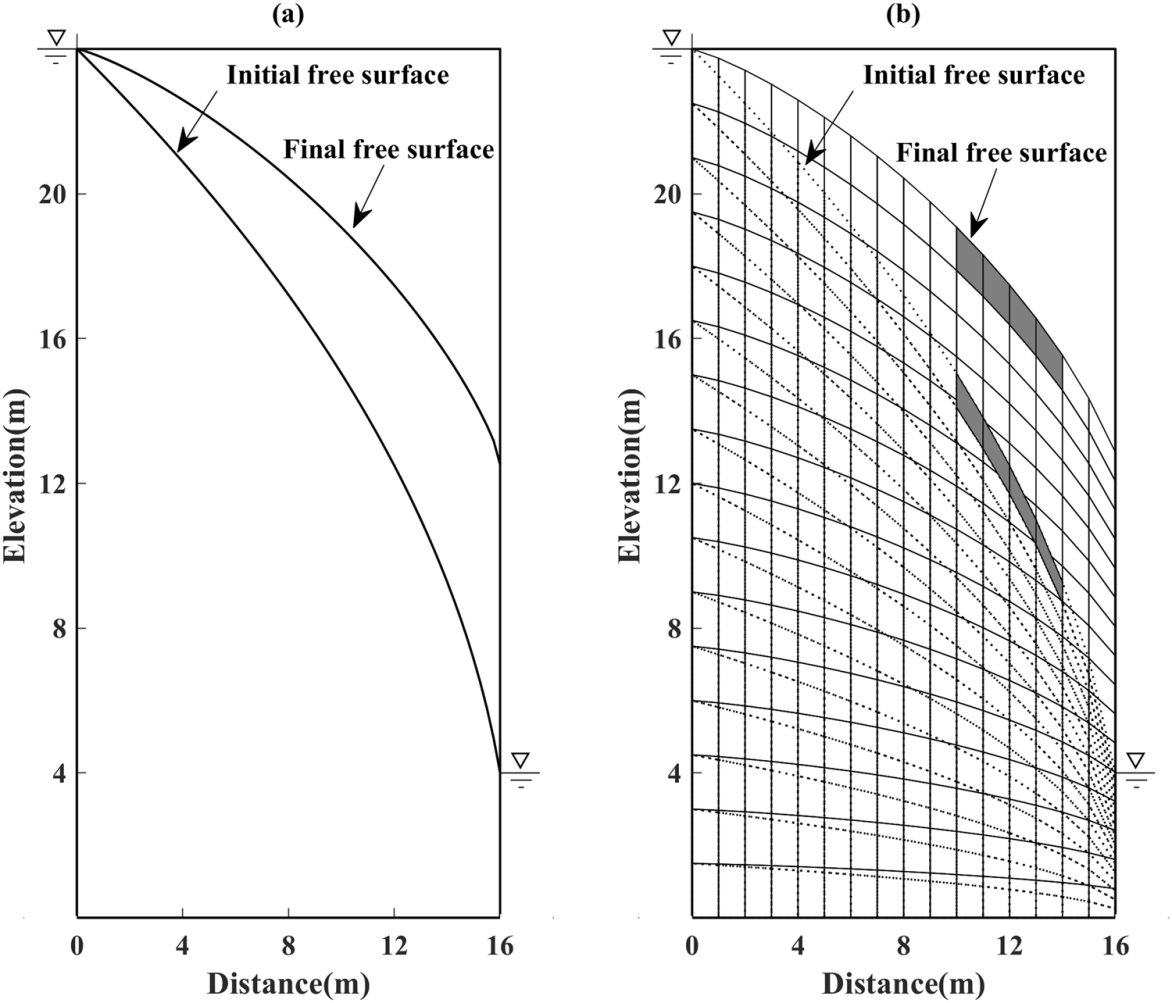
(**a**) The final free surface calculated by the proposed method and the initial free surface for Model 1 (left). (**b**) The meshing of the calculation domain for the initial free surface and final free surface for Model 1 (right).

As a moving mesh method is adopted to locate the free surface, the position and the shape of the element will change dynamically during every iteration. The factor *ζ* is set, in particular, to control the movement direction of the nodes on the free surface (e.g. vertically ascending or descending). Figure 6a shows the position of the initial free surface and the final free surface. Figure 6b shows the distribution of elements at the beginning and in the final iteration step. The dashed lines represent the initial distribution, and the solid lines represent the final calculated distribution. In order to highlight the difference, three shadowed grids are marked in the initial meshes, which are located on the initial free surface near the vicinity of the downstream boundary. As demonstrated in Fig. 6b, there is obviously a drastic change in the shape, size, and position of the shadowed grids.

**Figure 7:**
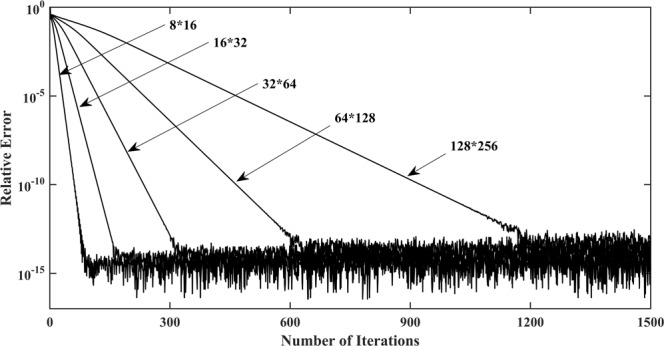
Impact of mesh fineness on the convergence rate and the relative error for Model 1.

**Figure 8:**
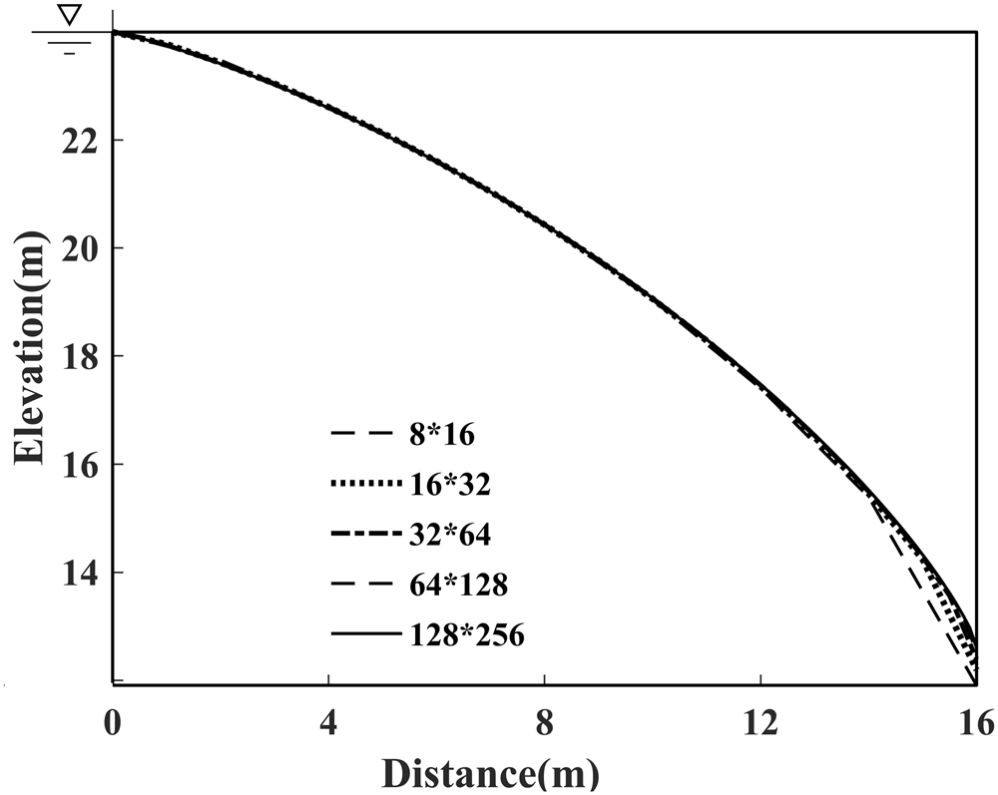
Impact of mesh fineness on the location of the free surface for Model 1.

Figure 7 shows the impact of mesh fineness on the convergence rate and the relative error. This calculation is performed by five different mesh fineness values to obtain different resolutions with 1500 iterations: 8 × 16 grids, 16 × 32 grids, 32 × 64 grids, 64 × 128 grids, and 128 × 256 grids, respectively. Taking the 8 × 16 grid case for example, as shown in Fig. 8, the calculation process converges to a steady state when it reaches roughly the 300th iteration, which is approximately 4 times the convergence rate of that of the 128 × 256 grid case. It seems that the method runs faster with coarser grids. Meanwhile, the location of the free surface closely approximates that of the fine grids. Nonetheless, the location of the exit point turns out to be lower, as shown in Fig. 8, which mainly resulted from the fact that, the position of the exit point is strongly related to the former node. From the presented results, it can be concluded that the coarser grids cannot meet the requirements for computational precision, and the proposed methodology combining the adaptive mesh strategy and Galerkin FEM with finer grids is more sufficient and accurate for the free surface search.

**Figure 9:**
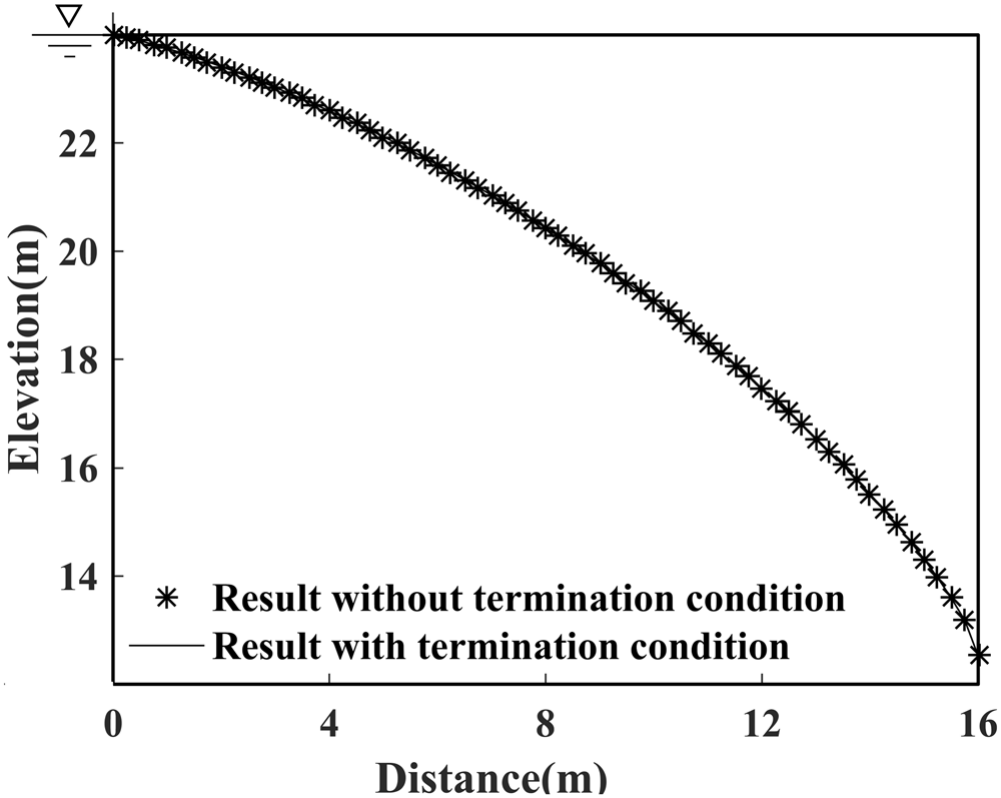
The calculated results with and without termination condition added to Model 1.

No matter what kind of mesh fineness is chosen, the minimum relative error occurs at approximately the 1300th iteration, as shown in Fig. 7. Moreover, it is worthy to note that the curve of the relative error exhibits strong fluctuations over a fixed interval near the position where the minimum relative error occurs. On account of its balance between efficiency and accuracy, a 64 × 128 grid case is again chosen to validate the effectiveness of the proposed termination condition. As far as the accuracy is concerned, Fig. 9 shows that the calculated result using a termination condition remains almost unchanged, which suggests that the precision is not affected by the chosen termination condition. For this reason, the selected termination condition is proven to be valid and feasible.

**Figure 10:**
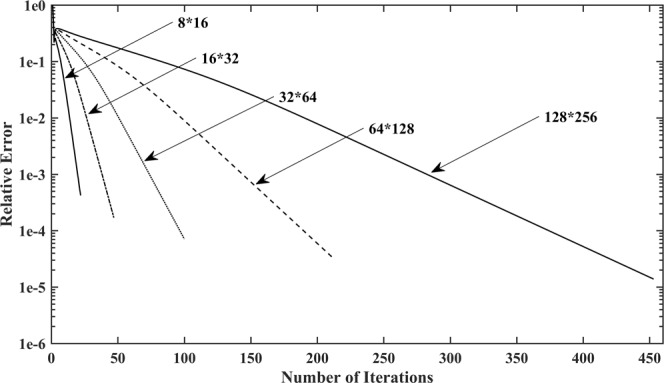
Impact of appropriate termination conditions on the convergence behaviour of Model 1.

Furthermore, the selected termination condition accelerates the convergence rate of the free surface search, as comparison results demonstrate in Fig. 8 and Fig. 10. The total number of iterations needed to reach a steady state with the termination condition added, take the 64 × 128 grid case for example, is considerably reduced from 610 to 220, by which we conclude that the choice of termination condition brings about a great reduction in calculation costs, and an excellent improvement in algorithm performance.

### Model 2, the second standard model

**Figure 11:**
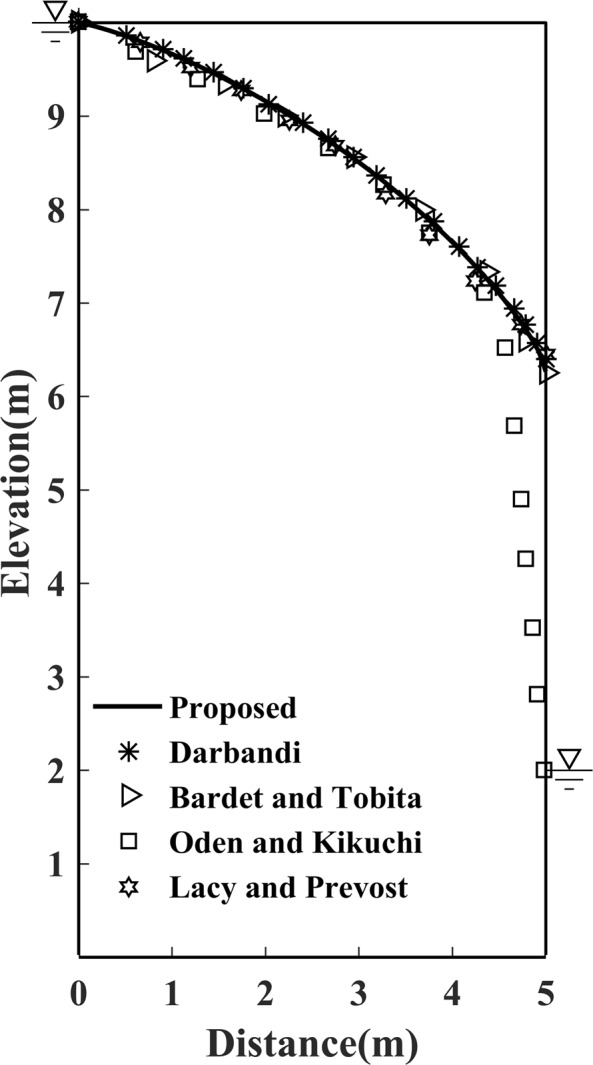
Free surface solution by proposed method to Model 2.

Similarly, for efficiency and accuracy consideration, as validated in the former example of Model 1, a 64 × 128 grid mesh distribution is again chosen. As shown in Fig. 11, except for the result by Oden^24^, the determined free surface with the proposed method is in good agreement with results provided by Lacy and Prevost^25^; Bardet and Tobita^17^ and Darbandi *et al*.^19^.

### Model 3, the Irregular model

**Figure 12:**
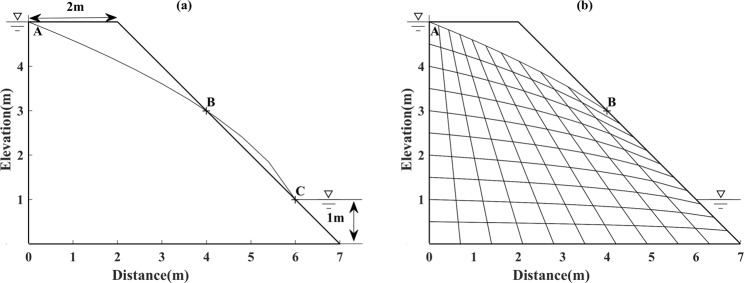
(**a**) The initial free surface intersects with the downstream boundary of Model 3 (left). (**b**) The initial free surface selection and grid subdivision schemes for Model 3 (right).

For this irregular model, analogously, the Dupuit assumption is first applied to determine the initial free surface. Nonetheless, the calculated result (i.e. curve **AC**) bends beyond the downstream boundary, as shown in Fig. 12a, which may lead to an incorrect search result. Therefore, another substitutional boundary needs to be implemented. Herein, the curve **AC** is substituted by the curve **AB** as the initial free surface for calculation, which is shown in Fig. 12b.

**Figure 13:**
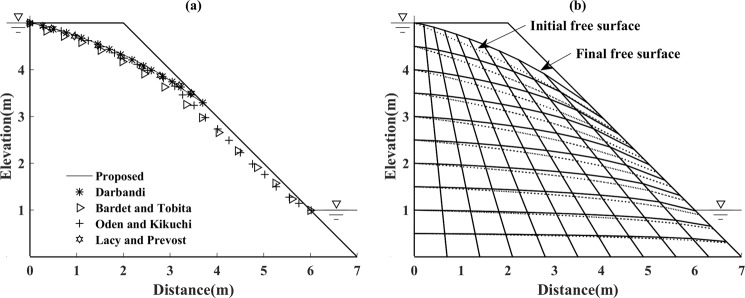
(**a**) Solution for free surface search by proposed method for Model 3 (left). (**b**) The initial free surface and the final free surface in the seepage domain for Model 3 (right).

The comparison results are shown in Fig. 13a, which presents good coincidence with the other four methods. Most importantly, one can observe that due to the fact that the height and width of every element is dynamically changing, accordingly, the nodes of the free surface move along a set of straight lines with a different fixed slope, as shown in Fig. 13b. The result of the calculation accuracy by the proposed method is not affected by the geometrical shape of model change, which validates the suitability and effectiveness of the present method for free surface search in the irregular geometric model.

### Model 4, the complex inhomogeneous model

This classic model, proposed by Oden and Kikuchi^24^, has been used as a benchmark by many researchers to test the accuracy of their numerical methods when drastic singularity exists on the free surface.

**Figure 14:**
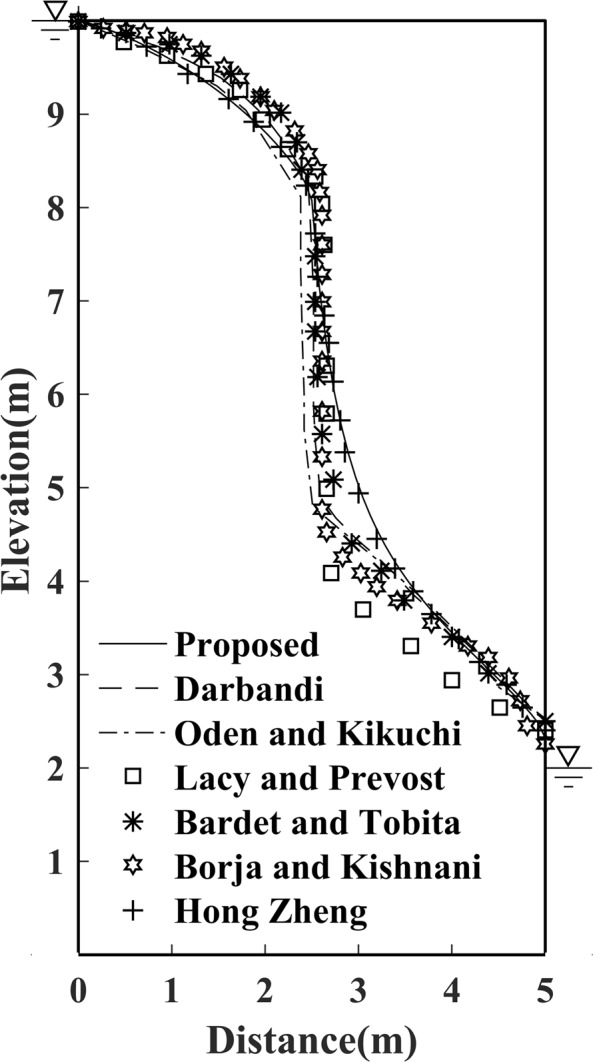
The current result by the proposed method for Model 4.

In this experiment, we also compare the results of the proposed method with those of traditional methods. With the same initial free surface chosen, and was as done for Model 2, the results of the free surface calculated by different methods are shown in Fig. 14.

Even though there is no analytical solution available for reference regarding this model, the result calculated by the proposed method shows good agreement with that by Zheng *et al*.^26^, who validated the correctness of results by utilizing the fact (i.e. flow conservation) that the rate of flow through distinct vertical sections in the dam should be equal to each other.

In this inhomogeneous model, because the Galerkin method based on the weak formulation is employed in the proposed algorithm, as well as that by Zheng *et al*.^26^, to discretize the control equations, an excellent effect for treating the drastic singularity of the free surface is guaranteed. Conversely, a strong solution which applies both the FDM and FVM may lead to a sharp discontinuity at the interface of the two blocks.

From this, it can be also concluded that, compared to the method of Zheng *et al*.^26^, the proposed method avoids the optimization experiments to determine R value (the radius of the influence domain of *k*th *ϕ* − node) and S value (the span of *ϕ* − node), as an inappropriate R and S value will affect the bandwidth of the matrix K, as well as the condition number of matrix K. As a result, the proposed method has characterized with simple programming, high computational efficiency, and excellent fitting accuracy, which is more sufficient and suitable for a complex inhomogeneous model with different permeability distributions.

## Conclusion

In this work, unconfined seepage problems were studied in four models with different complexities. The calculation process for the free surface solution is investigated with the standard model, irregular geometric model, and complex inhomogeneous media model. The major conclusions from this study are listed as follows:

As the most crucial part of the seepage problem is the precise confirmation of free surface position and the exit point location, the Dupuit assumption is employed to determine the initial free surface, by introducing two novel factors *ζ*, *λ* (i.e. the remainder factor of the normal vector and the step-size factor, respectively), the mesh can be dynamically reshaped to match the new location of the free surface during the next iteration, and subsequently, the accurate location of the exit point can eventually be fitted. Hence, it can be concluded that given the proper mesh fineness, the proposed algorithm shows better performance on the minimum error of free surface and exit-point position.

As ascertained above, if the search algorithm is run without any terminal condition, oscillating errors may occur, and subsequently, the errors accumulate over the entire iteration process which may lead to a waste in computing resources, or even worse, the accuracy may not improve. As a new solution, an innovative terminal condition was introduced, and the simulation result demonstrates that the number of iterations required to converge to a steady state in 64 × 128 grid case of Model 1 is considerably reduced from 610 to 220, which could bring about a great reduction in calculation costs, and a significant improvement in algorithm performance in free surface search.

An irregular grid technique was employed to fit the discrete domain of the irregular model. By virtue of smoothly fitting the data with this technique, the quality of mesh generation could be considerably improved. The simulated result of Model 3 shows that the proposed method is not affected by the change in the geometrical shape of the model, which validates the suitability and effectiveness of the application of an irregular geometric model.

For a more complex model case, in particular, our experiment shows that, by virtue of implementation of combining the weak formulation of Galerkin FEM with the AMM, the proposed algorithm achieves a better trade-off between efficiency and accuracy in dealing with inhomogeneous models with a sharp variation in the permeability distribution, which could provide a practical solution for the free surface search with complex models.

## Methods

### Free surface of unconfined seepage problem

**Figure 15:**
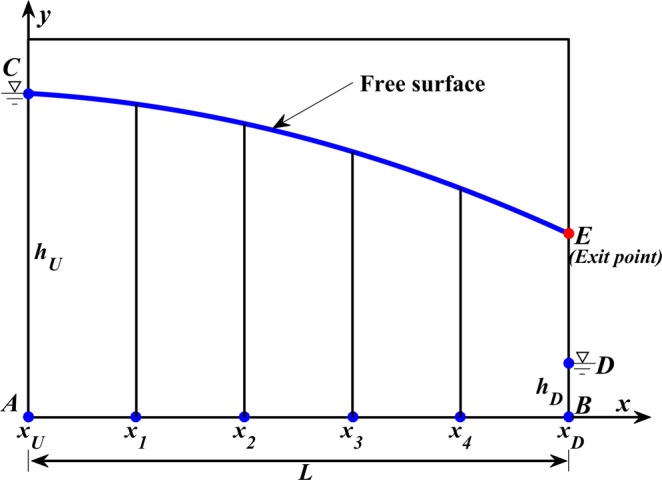
Schematic diagram of seepage flow.

Free surface seepage problems can be described as a generic condition, which is shown in Fig. 15, where zone ***ABDEC*** represents the saturated seepage domain^17^.

where *h*_*U*_ and *h*_*D*_ represent the total heads at upstream and downstream boundaries, respectively. ***ED*** is the seepage face, which means that when water seeps out of the dam the total head is equal to the elevation head because the pressure head is equal to zero on this face. ***L*** represents the length of the dam^27^.

The governing equation and boundary conditions of the free surface can be expressed as follows:

1 $$h(x,y)|{{\rm{\Gamma }}}_{1}={h}_{0}(x,y)\,({h}_{0}={h}_{U})\,{\rm{on}}\,{\rm{boundary}}{\boldsymbol{AC}}\,{\rm{or}}\,{h}_{D}\,{\rm{on}}\,{\rm{boundary}}\,{\boldsymbol{BD}}$$

2 $$\frac{\partial h}{\partial n}|{{\rm{\Gamma }}}_{2}=0\,({\rm{on}}\,{\rm{boundary}}\,{\boldsymbol{AB}})$$

3.1 $$h(x,y)|{{\rm{\Gamma }}}_{{\rm{3}}}=y(x)\,({\rm{on}}\,{\rm{boundary}}\,{\boldsymbol{CE}})$$

3.2 $$k\frac{\partial h}{\partial n}|{{\rm{\Gamma }}}_{3}=0\,{\rm{or}}\,{\bf{n}}\cdot {\bf{v}}=0\,({\rm{on}}\,{\rm{boundary}}\,{\boldsymbol{CE}})$$

4 $$h(x,y)|{{\rm{\Gamma }}}_{4}=y(x)\,({\rm{on}}\,{\rm{boundary}}\,{\boldsymbol{ED}})$$

where **n** denotes the normal direction.

### Accuracy simulation using Galerkin FEM method

As the finite element equation of 2D seepage is a partial differential equation, the boundary conditions and initial conditions must be satisfied for the solution to the partial differential equations. The derivation of the Galerkin method automatically satisfies the Neumann boundary conditions, namely, the derivative of an unknown function has a known value on the boundary. Hence, the Galerkin method is employed to deduce the control equation of the seepage problem.

Making use of the trial function, it yields:

5 $${\iint }_{{\rm{\Omega }}}\phi [\frac{\partial }{\partial x}({k}_{x}\frac{\partial h}{\partial x})+\frac{\partial }{\partial y}({k}_{y}\frac{\partial h}{\partial y})]d{\rm{\Omega }}=0$$

The first and second item of equation 5 can be transformed by the Green formula transformation:

6 $$\{\begin{array}{c}{\iint }_{{\rm{\Omega }}}\phi \frac{\partial }{\partial x}({k}_{x}\frac{\partial h}{\partial x})d{\rm{\Omega }}={\oint }_{{\rm{\Gamma }}}\phi ({k}_{x}\frac{\partial h}{\partial x}){n}_{x}d{\rm{\Gamma }}-{\iint }_{{\rm{\Omega }}}\frac{\partial \phi }{\partial x}({k}_{x}\frac{\partial h}{\partial x})d{\rm{\Omega }}\\ {\iint }_{{\rm{\Omega }}}\phi \frac{\partial }{\partial y}({k}_{y}\frac{\partial h}{\partial y})d{\rm{\Omega }}={\oint }_{{\rm{\Gamma }}}\phi ({k}_{y}\frac{\partial h}{\partial y}){n}_{y}d{\rm{\Gamma }}-{\iint }_{{\rm{\Omega }}}\frac{\partial \phi }{\partial y}({k}_{y}\frac{\partial h}{\partial y})d{\rm{\Omega }}\end{array}$$

According to the boundary conditions given above, the boundary integral of the first item on the right side of equation 6 is equal to zero. Substituting equation 6 into equation 5, it yields:

7 $${\iint }_{{\rm{\Omega }}}(\frac{\partial \phi }{\partial x}{k}_{x}\frac{\partial h}{\partial x}+\frac{\partial \phi }{\partial y}{k}_{y}\frac{\partial h}{\partial y})d{\rm{\Omega }}=0$$

As shown by equation 7, the weak solution of the control equation is obtained for the seepage field.

**Figure 16:**
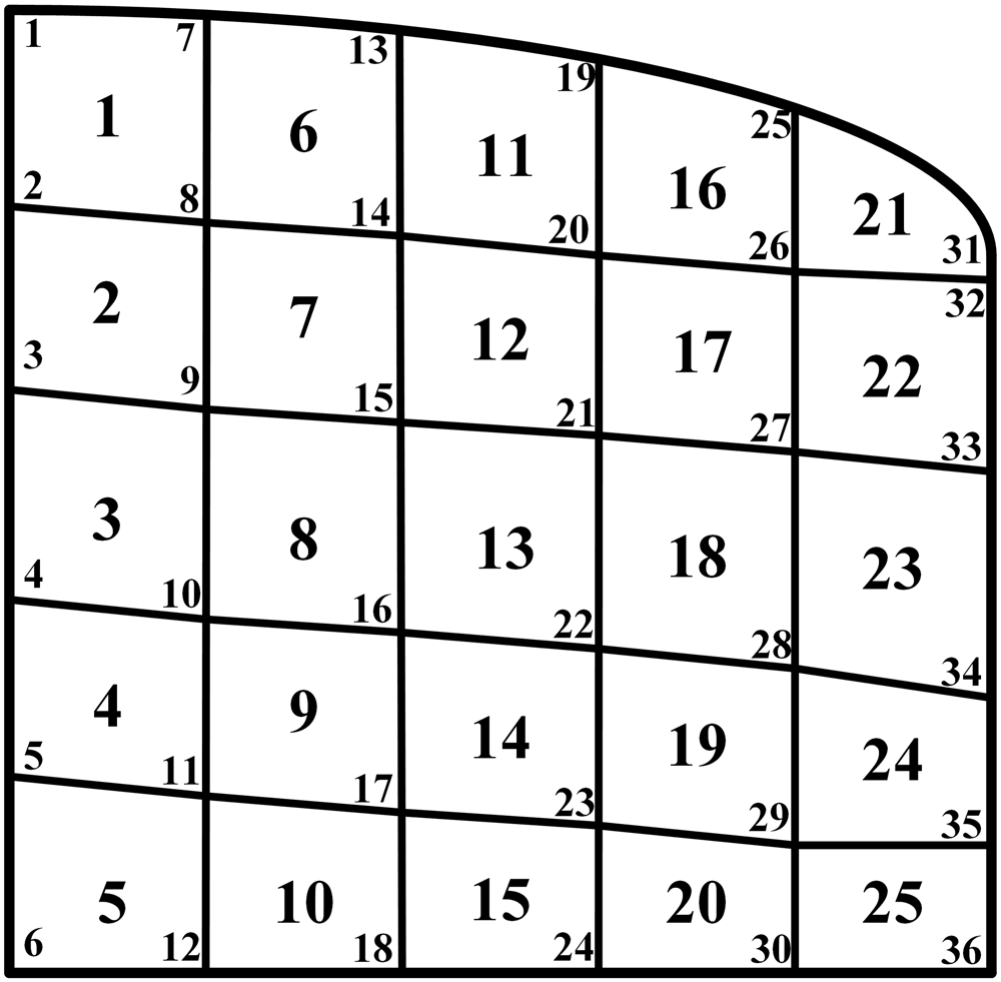
Schematic diagram of mesh subdivision and its node number for seepage field.

To clearly demonstrate the seepage field, the solution area is separated by irregular quadrangle grids, as shown in Fig. 16.

**Figure 17:**
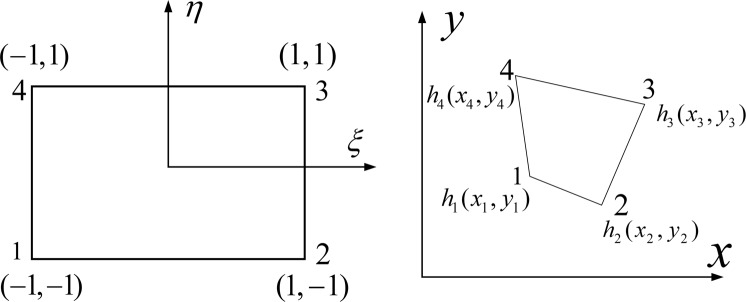
Schematic diagram of (**a**) Quadrilateral master element in *ξη*-plane (left). (**b**) Quadrilateral element in *xy*-plane (right).

The evaluation of element matrices and vectors using bilinear quadrilateral elements. The governing interpolation functions for a bilinear quadrilateral element are given by^28^:

8 $$\begin{array}{rcl}{N}_{1} & = & \frac{1}{4}(1-\xi )(1-\eta ),{N}_{2}=\frac{1}{4}(1+\xi )(1-\eta )\\ {N}_{3} & = & \frac{1}{4}(1+\xi )(1+\eta ),{N}_{4}=\frac{1}{4}(1-\xi )(1+\eta )\end{array}$$

Using an isoparametric representation, the *x*, *y* space coordinates of a point inside a quadrilateral and the primary unknown quantity are expanded in terms of the same interpolation functions, i.e. $x=\sum _{i=1}^{4}{x}_{i}^{e}{N}_{i}$, $y=\sum _{i=1}^{4}{y}_{i}^{e}{N}_{i}$ and $h=\sum _{i=1}^{4}{h}_{i}^{e}{N}_{i}$, where ${x}_{i}^{e},\,{y}_{i}^{e}$ for *i* = 1, 2, 3, 4 are the node coordinates of the quadrilateral element, and ${h}_{i}^{e}$ for *i* = 1, 2, 3, 4 are the values of the primary unknown quantity at the four nodes, as shown in Fig. 17.

Using the Jacobian transformation determinant, the differential relation of the two units can be expressed as follows:

9 $$dxdy=|\begin{array}{cc}\frac{\partial x}{\partial \xi } & \frac{\partial y}{\partial \xi }\\ \frac{\partial x}{\partial \eta } & \frac{\partial y}{\partial \eta }\end{array}|d\xi d\eta =|J|d\xi d\eta $$

The Jacobian transformation determinant |*J*| can be expressed as:

10 $$J=[\begin{array}{cc}\frac{\partial x}{\partial \xi } & \frac{\partial y}{\partial \xi }\\ \frac{\partial x}{\partial \eta } & \frac{\partial y}{\partial \eta }\end{array}]=[\begin{array}{cccc}\frac{\partial {N}_{1}}{\partial \xi } & \frac{\partial {N}_{2}}{\partial \xi } & \frac{\partial {N}_{3}}{\partial \xi } & \frac{\partial {N}_{4}}{\partial \xi }\\ \frac{\partial {N}_{1}}{\partial \eta } & \frac{\partial {N}_{2}}{\partial \eta } & \frac{\partial {N}_{3}}{\partial \eta } & \frac{\partial {N}_{4}}{\partial \eta }\end{array}]\,[\begin{array}{cc}{x}_{1} & {y}_{1}\\ {x}_{2} & {y}_{2}\\ {x}_{3} & {y}_{3}\\ {x}_{4} & {y}_{4}\end{array}]$$

Substituting equation 8 into equation 10, we obtain:

11 $$|J|=\frac{1}{16}|\begin{array}{cc}\alpha \eta +{c}_{1} & \beta \eta +{c}_{2}\\ \alpha \xi +{c}_{3} & \beta \xi +{c}_{4}\end{array}|=A\xi +B\eta +C=J(\xi ,\eta )$$

where

$$\begin{array}{c}A=(\beta {c}_{1}-\alpha {c}_{2})/16,\,B=(\alpha {c}_{4}-\beta {c}_{3})/16,\,C=({c}_{1}{c}_{2}-{c}_{2}{c}_{4})/16\\ \alpha =-{x}_{1}+{x}_{2}-{x}_{3}+{x}_{4},\,\beta =-{y}_{1}+{y}_{2}-{y}_{3}+{y}_{4},\,{c}_{1}=-{x}_{1}-{x}_{2}+{x}_{3}+{x}_{4}\\ {c}_{2}=-{y}_{1}-{y}_{2}+{y}_{3}+{y}_{4},\,{c}_{3}={x}_{1}-{x}_{2}-{x}_{3}+{x}_{4},\,{c}_{4}={y}_{1}-{y}_{2}-{y}_{3}+{y}_{4}\end{array}$$

Applying the Galerkin method in equation 10, a new function **N** is introduced and treated as a basic function for expanding and testing. The seepage in the cells can be expressed as *h*^*e*^(*x*, *y*) = **Nh**^**e**^, substituting this expression into equation 7, the ordinary differential equations of the seepage field equations can be obtained:

12 $$\sum _{e=1}^{NE}{{\bf{A}}}^{e}{{\bf{h}}}^{e}=0$$

where *NE* represents the total number of elements, **h**^*e*^ represents the seepage head of the unit, and the matrix **A**^*e*^ can be expressed as:

13 $${{\bf{A}}}^{e}={{\bf{A}}}^{ex}+{{\bf{A}}}^{ey}={\iint }_{e}({k}_{x}\frac{\partial {{\bf{N}}}^{T}}{\partial x}\frac{\partial {\bf{N}}}{\partial x}+{k}_{y}\frac{\partial {{\bf{N}}}^{T}}{\partial y}\frac{\partial {\bf{N}}}{\partial y})dxdy$$

Substituting the differential expression into the corresponding items in equation 13 yields:

14 $${{\bf{A}}}^{eij}={{\bf{A}}}^{exij}+{{\bf{A}}}^{eyij}={\int }_{-1}^{1}{\int }_{-1}^{1}\frac{{k}_{x}{F}_{ix}(\xi ,\eta ){F}_{jx}(\xi ,\eta )+{k}_{y}{F}_{iy}(\xi ,\eta ){F}_{jy}(\xi ,\eta )}{|J|}d\xi d\eta $$

where ${F}_{ix}(\xi ,\eta )=\frac{\partial y}{\partial \eta }\frac{\partial {N}_{i}}{\partial \xi }-\frac{\partial y}{\partial \xi }\frac{\partial {N}_{i}}{\partial \eta }$, and ${F}_{iy}(\xi ,\eta )=-\frac{\partial x}{\partial \eta }\frac{\partial {N}_{i}}{\partial \xi }+\frac{\partial x}{\partial \xi }\frac{\partial {N}_{i}}{\partial \eta }$, which can be obtained by equation 8 and equation 10, respectively. Hence, the element matrix can be obtained by equation 14 using Gaussian numerical integral.

By expanding the unit column vector **h**^*e*^ to the column vector **h** of all nodes, as well as expanding the unit matrix **A**^*e*^ to matrix **A** of the whole region and merging the elements, the finite element equation of the whole seepage solution domain can be obtained:

15 $${\bf{A}}{\bf{h}}=0$$

where **A** represents the stiffness matrix, and **h** represents the total head vector.

In fact, the Dirichlet boundary condition is generally determined by the multiplied bigger number method (MBNM) using FEM^29^. The MBNM has proven to be the most efficient and straightforward technique in solving a stiffness matrix. However, it does not allow the elimination of equations associated with the nodes on the boundary, which may result in an increase in the number of conditions and consequently, an increase in the burden of iterations^29^. To solve this problem, a classic method, named direct method (DM), is introduced to impose the boundary conditions which reduce the order of matrix, simplify the process of iteration, and re-encode the unknown node.

The total coefficient matrix can be divided into two parts, yielding:

16 $$[\begin{array}{cc}{{\bf{A}}}_{bb} & {{\boldsymbol{{\rm A}}}}_{bf}\\ {{\bf{A}}}_{fb} & {{\bf{A}}}_{ff}\end{array}]\,[\begin{array}{c}{{\bf{h}}}_{b}\\ {{\bf{h}}}_{f}\end{array}]=0$$

where *b* represents the node number of the known boundary, and *f* represents the node number of unknown grids.

The flow values on the boundary can be determined by equations 1–4.

Expanding equation 16 yields:

17 $${{\bf{A}}}_{ff}{{\bf{h}}}_{f}=-{{\bf{A}}}_{fb}{{\bf{h}}}_{b}$$

### Adaptive strategy for free surface analysis

To determine an initial seepage model, an initial free seepage value needs to first be confirmed. The saturated domain is comprised of zone ABDEC, as shown in Fig. 15, where the location of free seepage (i.e. curve CE) is unknown.

Assuming the media that seepage flows through is homogeneous and isotropic, the location of the initial free surface (i.e. curve CE) can be obtained by the Dupuit assumption^30^:

18 $$y=\sqrt{{H}_{1}^{2}-\frac{x}{L}({H}_{1}^{2}-{H}_{2}^{2})}$$

where *H*_1_, *H*_2_ represent the upstream water head and downstream water head, respectively. *L* represents the length of curve AB.

As mentioned above, equations 1–4 are the required boundary conditions that restrain the calculation, equation 1 is employed to define the water head at the left boundary (i.e. curve AC) and right boundary (i.e. curve BD). If the boundary ED appears as a part of our solution domain boundaries, equation 4 can be utilized to specify the water head on the boundaries. Meanwhile, equation 3.1 can be used to implement the required boundary condition at the free surface. As the condition of free surface needs to be modified during each iteration, the speed of free seepage in normal direction must satisfy equation 3.2.

**Figure 18:**
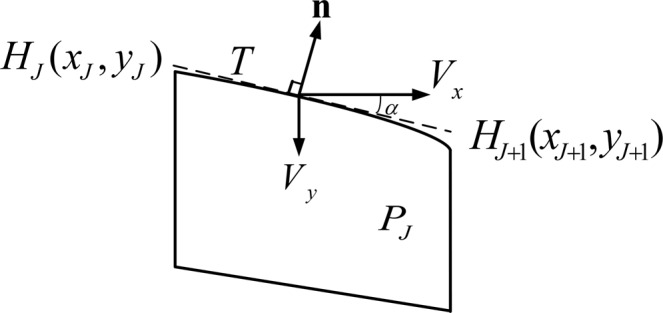
A boundary element on the free surface.

Assuming cell *P*_*J*_ is a boundary element on the free surface, as shown in Fig. 18, the boundary condition can be written as:

19 $${\bf{n}}\cdot {\bf{v}}={\bf{n}}\cdot [-{\bf{K}}\nabla (h)]={k}_{x}\frac{\partial h}{\partial x}(\sin \,\alpha )-{k}_{y}\frac{\partial h}{\partial y}(\cos \,\alpha )=0$$

where line *T* represents the tangent line.

As the location of the initial free surface may not be accurate at this stage, the boundary condition of equation 19 is not equal to zero. Considering a non-zero gradient on the left side of equation 19, a novel remainder factor *ζ* is introduced to determine the normal vector of the elements on the free surface. In this regard, the parameter *ζ* can be defined as:

20 $${\zeta }_{J}={[{k}_{x}\frac{\partial h}{\partial x}(\sin \alpha )-{k}_{y}\frac{\partial h}{\partial z}(\cos \alpha )]}_{J}$$

where *J* denotes the *J*-*th* element on the free surface. If ${\zeta }_{J} > {\rm{0}}$, it implies a node above the free surface. On the contrary, the case ${\zeta }_{J} < {\rm{0}}$ implies a node within the saturated domain. The factor *ζ* provides a general judgment criterion to determine the movement direction of the node on the free surface. The iterations will be continued until the factor *ζ* less than a tolerable error. Therefore, the magnitude of factor *ζ* is employed to relocate to a new position on the free surface:

21 $${h}_{J}^{i+1}={h}_{J}^{i}+\lambda {\zeta }_{J}$$

where *i* denotes the number of iterations, and *λ* denotes the step-size parameter which is specially designed and capable of controlling the iteration speed.

In the iteration process, although a large value *λ* here can guarantees a high convergence rate, it will cause oscillation and thus cannot achieve the specified tolerance. In contrast, although a small large value *λ* represents a higher precision solution, it might indicate an increasement of the iterations and deceleration of the convergence rate. Hence, an appropriate step-size parameter should be determined according to the model size, computation efficiency and calculation accuracy. For the models in the present work, the experience and test process showed that *λ* = 1.28*L/(*NX* − 1) (where *L* represents the length of earth dam, *NX* represents the number of nodes in *x* direction) would provide a suitable convergence behaviour.

### Tolerable error and termination condition

Due to the boundary condition requirements that the free surface needs to meet, note that a specified tolerance with respect to the magnitude of *ζ* is unavoidable. Hence, a tolerable error *ψ* is introduced specifically to determine whether the free surface meets the boundary conditions or not, and which will lead to great improvement in saving computational resources and high efficiency in the search process. Equation 3.2 can be rewritten and a termination condition is defined as:

22 $$\zeta \le \psi $$

where tolerable error *ψ* is defined as:

23 $$\psi ={\rm{0.001}}/N$$

where *N* represents the number of grids in the *x*-direction.

In the case where equation 22 is workable, we can confirm that the correct and accurate position of the free surface has been determined.

## Supplementary information

Dataset 1
